# Enhanced Automated Guidance System for Horizontal Auger Boring Based on Image Processing

**DOI:** 10.3390/s18020595

**Published:** 2018-02-15

**Authors:** Lingling Wu, Guojun Wen, Yudan Wang, Lei Huang, Jiang Zhou

**Affiliations:** 1 School of Mechanical & Electronic Information, China University of Geosciences, Wuhan 430074, China; 13951211537@163.com (L.W.); wangyodan@163.com (Y.W.); zhoujiangmvp@163.com (J.Z.); 2 Shandong Institute of Space Electronic Technology, Yantai 264670, China; huanghuanlei@163.com

**Keywords:** trenchless, image processing, guidance system, auto-focus

## Abstract

Horizontal auger boring (HAB) is a widely used trenchless technology for the high-accuracy installation of gravity or pressure pipelines on line and grade. Differing from other pipeline installations, HAB requires a more precise and automated guidance system for use in a practical project. This paper proposes an economic and enhanced automated optical guidance system, based on optimization research of light-emitting diode (LED) light target and five automated image processing bore-path deviation algorithms. An LED target was optimized for many qualities, including light color, filter plate color, luminous intensity, and LED layout. The image preprocessing algorithm, feature extraction algorithm, angle measurement algorithm, deflection detection algorithm, and auto-focus algorithm, compiled in MATLAB, are used to automate image processing for deflection computing and judging. After multiple indoor experiments, this guidance system is applied in a project of hot water pipeline installation, with accuracy controlled within 2 mm in 48-m distance, providing accurate line and grade controls and verifying the feasibility and reliability of the guidance system.

## 1. Introduction

Horizontal auger boring (HAB) is a common trenchless construction method for installing gravity or pressure pipes, including sewer and hot water pipelines. It has advantages of low disruptions to the environment, low footprint, small earthwork quantity, and less external interference [1,2,3,4]. Before drilling, two pits, a “jacking pit” and a “reception pit”, are excavated. A drilling rig is installed inside the jacking pit. During drilling, the rig pushes the pipelines towards the reception pit in the designated direction. The spoil is removed with helical screw conveyors [5]. Owing to differing soil conditions and the influence of gravity, the drilling track often skews. Therefore, a guidance system is required to monitor the line and grade in real time for taking corresponding control measures.

Common guidance for monitoring line and grade is divided into two categories. One is the electromagnetic method, and the other is the optical method. The electromagnetic method is comprised of a receiver, a display, and a transmitter. The transmitter is installed underground at the end of the drill bit, whereas the receiver is held by an operator on the ground. The system requires one operator to scan the ground, decreasing efficiency [6]. By this method, the monitoring error increases with the depth of the transmitter. The guidance precision barely meets the requirements of gravity and pressure pipelines. Additionally, electromagnetic signal is highly vulnerable to the underground pipes and geomagnetic field [7]. The optical methods have the advantage of high precision and unaffected by the geomagnetic field. They can be further divided into two parts, based on the optical target. Those are laser and LED guidance systems. Normally, a laser guidance system is employed when the project is large-diameter and long distance. The LED guidance system employs a target made of LEDs, used in small-diameter and short-distance projects. However, its guidance accuracy greatly depends on operator control, because one is required to observe the image using a charge-coupled device (CCD) camera and to manually estimate the deflection, resulting in an elusive operating standard [8]. 

This article presents an enhanced automated deflection detection method based on image processing to achieve high guidance system accuracy for HAB. Emphasis is placed on efforts to enhance image quality, including optimization of LED targets, image reprocessing and feature extraction. Further pixel calculations are applied to reprocessed images. The auto-focus algorithm maintains clarity of images. Additionally, concrete values of drill-bit deflection, face and rotation angles are calculated and displayed on a personal computer, so that the operators can be rid of manual drilling estimations. The proposed guidance system can overcome many limitations of the current manual evaluation and can improve the level of automation.

## 2. Components of the Enhanced Automated Guidance System 

**Figure 1:**
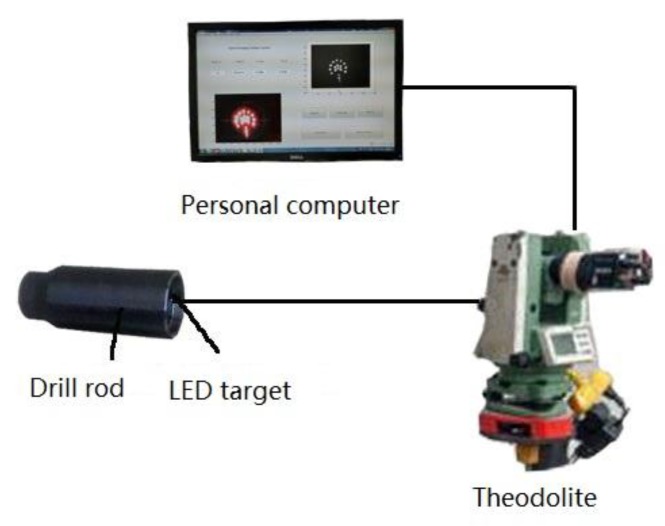
Main components of the proposed guidance system.

The enhanced automated guidance system consists of an LED target, a personal computer, and a theodolite, as shown in Figure 1. The LED target acts as the signal, following the drill bit. A CCD camera, mounted on the theodolite, shoots images of the LED target. The personal computer is employed to preprocess images, extract features, calculate angles and deflection and display results.

**Figure 2:**
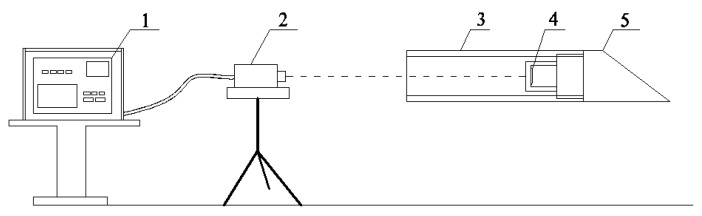
Relative position of components in the guidance system: 1, monitor; 2, charge-coupled device (CCD) camera; 3, drill rod; 4, light-emitting diode (LED) target; and 5, drill bit.

The components are set up as shown in Figure 2. The LED target (Figure 2, part 4), installed at the end of a drill bit (Figure 2, part 5), advances with the drill bit. The theodolite (Figure 2, part 2) is in the jacking pit, at the same grade with the center of the drill rod (Figure 2, part 3). It continues monitoring the LED target through the vacant drill string during drilling. At set intervals, images of LED target are shot and immediately transmitted to the personal computer (Figure 2, part 1). Subsequently these images are processed to obtain deflection and rotation angle, which are also displayed on the monitor.

## 3. Design and Optimization of the LED Target

**Figure 3:**
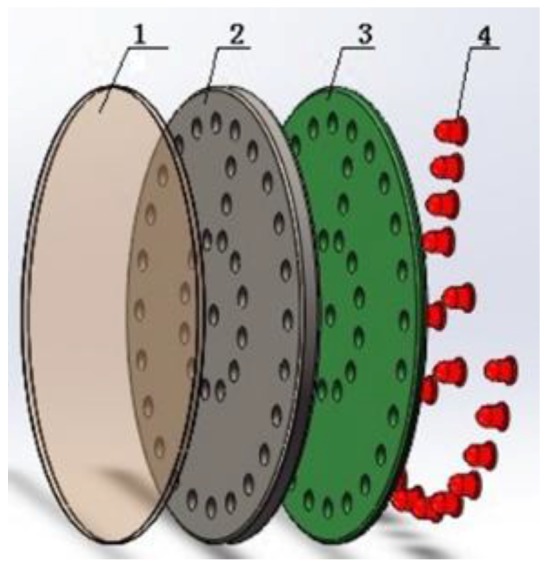
Constitution of designed LED target: 1, filter plate; 2, fixing plate; 3, printed circuit board (PCB); and 4, LEDs.

The LED target contains a filter plate, a fixing plate, a printed circuit board (PCB), and LEDs, as shown in Figure 3. The filter plate is employed to filter the LED light. The PCB is used to simplify the electro circuit. All LEDs are laid out in two circles and one long line. The two circles are used to show the center of the target, whereas the long line indicates direction. The target is customized for the drill rod with the inside diameter of 60 mm. Thus, the diameter is designed as 60 mm. 

### 3.1. Optimization of LED Target

LED target image clarity is very important in calculating deflection and drill-bit angle. The LED color, filter plate, light intensity, and distance between LEDs may all affect image quality. Therefore, experiments are conducted to optimize the LED target.

#### 3.1.1. LED Color Selection

The target is used in a dark environment. Thus, proper light color will benefit image quality. Short wavelength light is more easily scattered. Red light, common among LED colors, has the longest wavelength. Therefore, we use red LEDs to build the target. To ensure that the red LED works best, experiments are conducted with different colored targets (i.e., red, pink, yellow, blue, green, and white). Testing the images at 100 m, the red LEDs are clearest, as expected, for distinguishing the center and direction of the target. 

#### 3.1.2. Filter Plate Color Selection

To improve the clarity of the image, we install a filter plate in front of the LED target to improve light purity. Four filter plates (i.e., red, yellow, green, and blue) are fabricated and installed in front of the target. Checking the images at 100 m, the blue filter, combined with the red LED target, has the best image quality.

#### 3.1.3. Resistance Selection

Proper light intensity is important to image quality. LED light intensity can be changed with electro circuit resistance. Therefore, a slide rheostat is connected in series with the LED target. We change the slide rheostat every 20 Ω and observe the image. When resistance rests between 140 Ω to 180 Ω, the images are relatively clear. Therefore, we employ a resistance of 160 Ω on the electrical circuit.

#### 3.1.4. Distance between LEDs Selection

**Figure 4:**
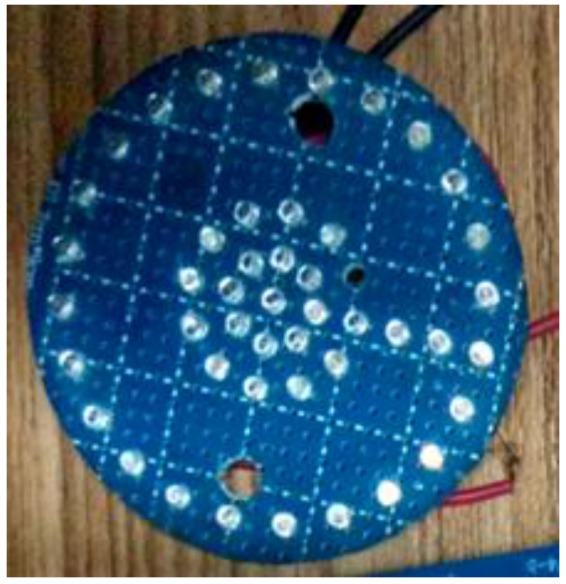
LED target for distance selection experiment.

The distance between LEDs affects image quality. If the distance is large, it is advantageous to distinguish every LED in the image while increasing the size of the target. Otherwise, the outline of each LED is too near to be distinguished. Therefore, we find the optimum distance between LEDs to clarify the outline of every diode. Figure 4 displays the LED target used in the distance selection experiment. The LEDs on the target are very densely installed. We successively turn off LEDs in different circles and intervals to contrast the outlines. As before, we evaluate the images at 100 m. When the distance between LEDs is 11 mm, the outline of every LED is the clearest. 

Based the experiments above, the parameters for the LED target are obtained. The target requires a 60-mm diameter, red LED, blue filter plate, 11-mm distance, 160-Ω resistance, and two 1.5-V dry batteries. 

### 3.2. Verification of LED Target

**Figure 5:**
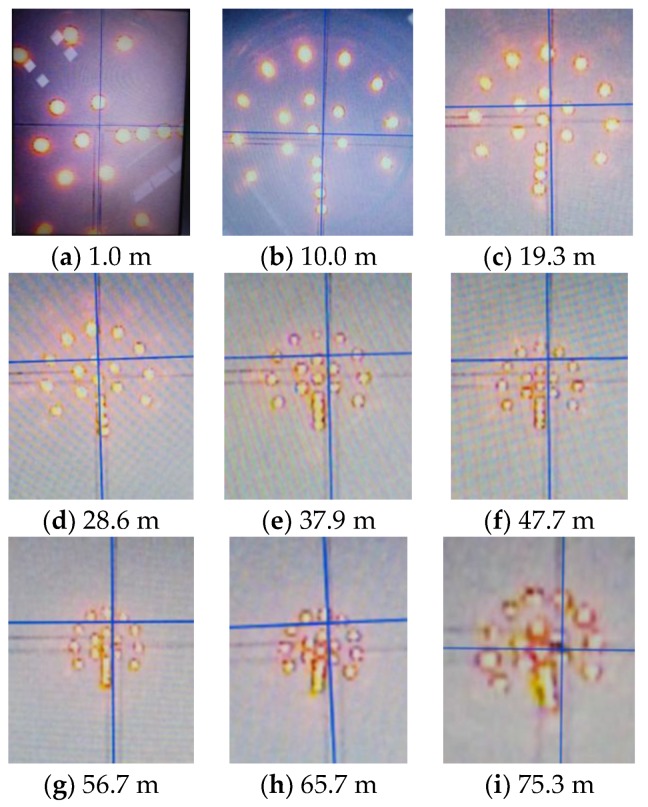

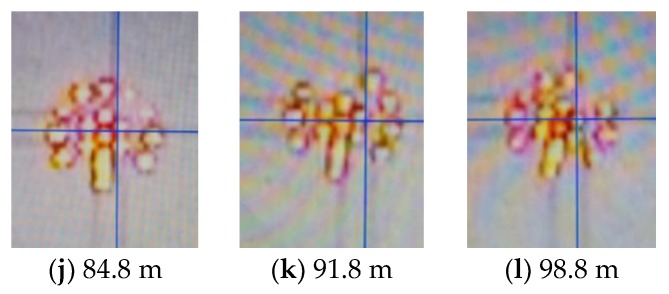
Images of LED target captured by CCD camera at different distances.

**Figure 6:**
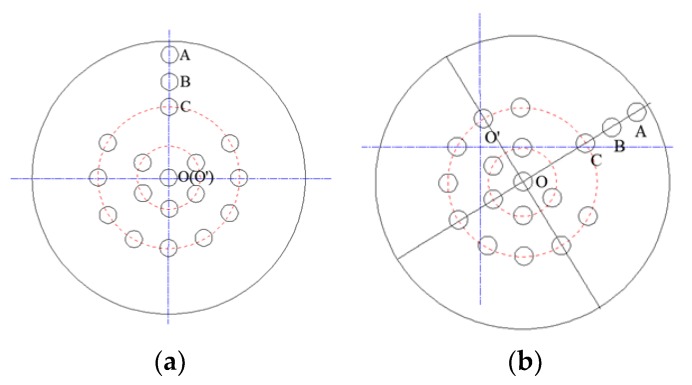
Schematic diagram of target state: (**a**) target coinciding with drill rod; (**b**) target deviating from drill rod (drill bit deviating from planned track). The blue reference lines are to show the center of the drill rod. The red lines are to show the layout of LEDs.

To verify the effect of the optimized LED target, an indoor experiment is conducted to shoot images. In this test, our optimized LED target is installed at the end of each drill rod, whose length is about 9 m. Figure 5 shows the images of the target shot by a CCD camera. Figure 5a is shot when the camera is installed 1 m from the drill string. Figure 5b is shot when the target is installed at the end of the first drill rod, and so forth. By checking the images in Figure 6, we can see that the outline of each LED, even those in the inner circle, is clearly distinguished within 47.7 m. Beyond that, the inner LEDs are hardly not able to be discerned. However, the outer ones are still clear enough to be discriminated. Additionally, LED images are still useful for pointing the direction, even at 98.8 m.

Our designed LED target, at long distance, has the following characteristics: simple fabrication, ease of installation, low cost, and good image quality.

## 4. Software System Design

For HAB, if the drill bit advances in the predesigned track, the center of the LED target (i.e., point *O*) coincides with the center of the drill rod (i.e., point *O′*), because these images are shot through a vacant drill string, as shown in Figure 6a. Otherwise, the drill rod and the LED target are not concentric, as illustrated in Figure 6b. Therefore, monitoring the target state is important to obtain the drilling situation.

Once deviation occurs, an operator must immediately stop boring and steer the drill bit a certain angle (i.e., rotation angle) to make the line, *OCBA*, run through point *O′*. Then, the forward direction can be changed by thrusting the drill bit, because it has a slanted face that enables it to move in any desired direction at any time [9]. Thrusting should be stopped only when point *O* meets point *O′*, meaning the drill bit returns to the pre-defined track. Thus, the rotation angles and deflection are significant for correction. In the image, they appear as the angle between line *OO′* and line *OCBA*, and the length of line *OO′*. 

**Figure 7:**
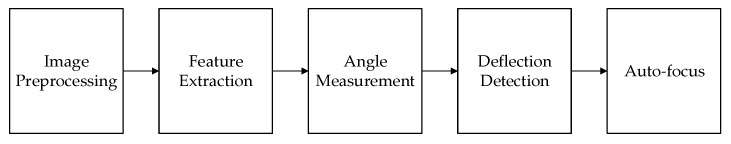
Software system design block diagram of image processing.

Image processing technology is the core of the software design, which can extract the characteristic information of the target. Image preprocessing is employed to obtain suitable images for feature extraction and pixel calculation (i.e., angle measurement and deflection detection). Additionally, auto-focus is achieved by mechatronics design based on the processed images. The software system design block diagram is given in Figure 7. All image processing is conducted in MATLAB (R2017b, MathWorks^®^, Natick, MA, USA).

### 4.1. Image Preprocessing Algorithm

**Figure 8:**
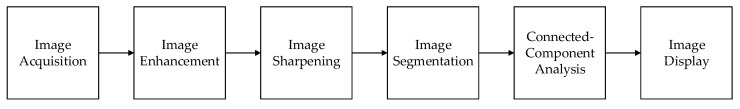
Flowchart of image preprocessing.

**Figure 9:**
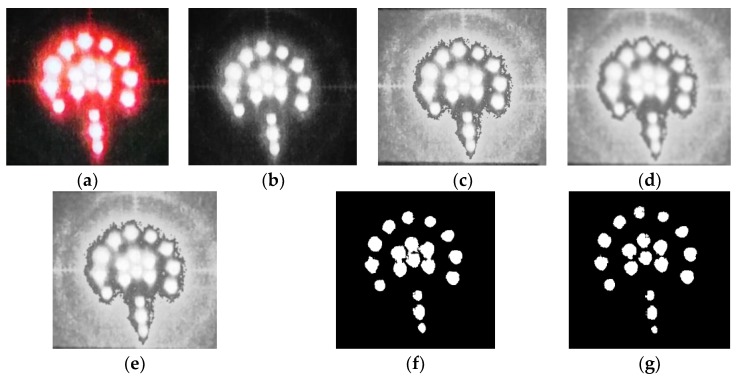
Target images in each processing procedure. (**a**) Original image; (**b**) Gray image; (**c**) Image after histogram equalization; (**d**) Smoothed image; (**e**) Sharpened image; (**f**) Binary image; (**g**) Final image.

Image preprocessing is important to promote the efficiency of feature extraction, pixel calculation (i.e., angle measurement and deflection detection) and auto-focus. The fowchart of image preprocessing is shown in Figure 8. Corresponding target images of each preprocessing procedure are shown in Figure 9.

The color image (Figure 9a) captured by the camera, term as original image, must be firstly converted to a grayscale image in order to reduce storage space and promote operation efficiency. The conversion from RGB to gray can be achieved using Equation (1).

(1) Y=0.3R+0.59G+0.11B

where *R*, *G*, *B* are the red, green and blue components of the RGB image respectively. Figure 9b shows the gray image after conversion.

Image enhancement can highlight the region-of-interest (ROI) contrast against the background. It also weakens or eliminates interference to make images more suitable for special applications [10,11,12]. To enhance LED target images, techniques, including gray histogram, image smoothing, and image sharpening, are implemented on images.

The histogram of a digital image can reflect an overview of this image. It is a practical and effective way to enhance the image using histogram equalization, which expands the selected range of intensity. The target image, processed by histogram equalization, is shown in Figure 9c.

After histogram equalization, image clarity improves. Concurrently, image noise increases. Image smoothing [13] can be used to compress image noise. In this paper, we choose the smoothing linear filters (i.e., averaging filters) for their real-time performance and reliable image quality, compared to several other linear and non-linear filters. Averaging filters can be noted by the expression (2).

(2) $$g(x,y)=\frac{1}{M}\sum_{(m,n)\in S}f(m,n)$$

where *M* is the total number of coordinate points within the collection, f(m,n) is the input image, g(x,y) is the processed image, *S* is a collection of coordinate points in the (x,y) domain, but the point (x,y) is not included. The post-smoothing image is shown in Figure 9d. 

Smoothing makes the image blurred again. Thus, image sharpening [14] is required to enhance edges and grayscale transitions. A Gaussian filter is a high-pass filter widely used to sharpen images. The transfer function of the *n*th Gaussian high-pass filter with cut-off frequency, $D_{0}$, is formulated as the Equation (3).

(3) $$H(u,v)=e^{-{\left[\frac{D_{0}}{D(u,v)}\right]}^{n}}$$

where $D_{0}$ is the cut-off frequency, $D(u,v)={\left[u^{2}+v^{2}\right]}^{\frac{1}{2}}$, and *n* controls the increase rate of H(u,v). Figure 9e shows the image after sharpening.

**Figure 10:**
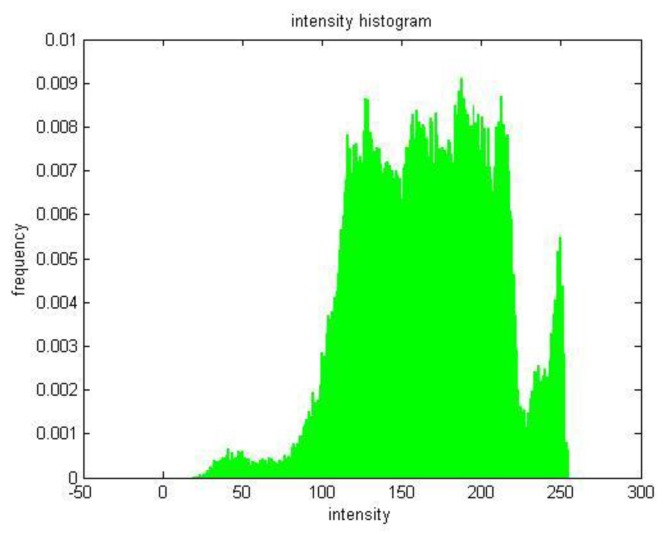
Intensity histogram of a sharpened image (Figure 6e).

Image segmentation is used to classify image features to highlight and extract ROIs [15]. Image thresholding enjoys a central position of image segmentation applications because of its intuitive properties and implementation simplicity. A thresholded image, g(x,y), is defined as the expression (4).

(4) $$g(x,y)=\left\{ \begin{array}{l} 0,f(x,y)<T\\ 1,f(x,y)\geq T \end{array}\right.$$

where f(x,y) is the input image, and T is the threshold. Pixels labeled 1 correspond to objects, whereas pixels labeled 0 correspond to the background. A conventional gray-level format with an 8-bit depth is employed here; the brightness range is 0–255 [16]. A proper threshold is crucial to image effect during threshold segmentation. The segmentation point is obtained with an analysis of intensity histogram. Figure 10 shows the intensity histogram of a sharpened target image. It contains three dominant modes, amongst which the brightest domain represents illuminating diodes in the target. There is an obvious valley of intensity, which can be chosen as a threshold to segment the image. In this paper, *T* = 235 and the outcome binary image is shown in Figure 9f.

After the binary image processed, connected-component analysis is applied to further separate objects using binary large object tool in MATLAB (R2017b, MathWorks^®^, Natick, MA, USA) [17]. The final image of preprocessing is shown in Figure 9g.

### 4.2. Feature Extraction Algorithm

**Figure 11:**
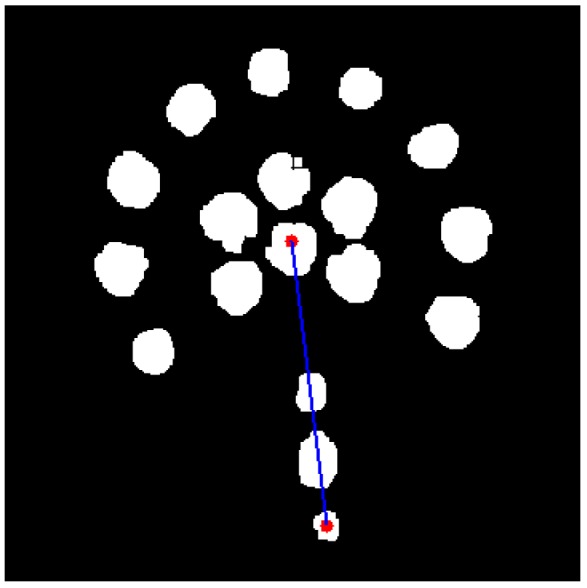
Target image with direction location. (The above red dot means the center of the target image while the below is the center of the direction point. The blue line joining there two points are to show the direction of drill bit.).

The points *O* and *A* in Figure 6 are features of target image whose extraction algorithm bases on calculation on pixels in the preprocessed image. Algorithms are programmed to sum up pixels of each connect-component and to calculate their mean value as the center point (i.e., point *O*) of the target image. To locate direction, the outermost component can be recognized as a direction point whose center is also calculated by average processing. Then a line joins these two points to precisely show the direction. The final processed result is shown in Figure 11. 

### 4.3. Angle Measurement Algorithm

**Figure 12:**
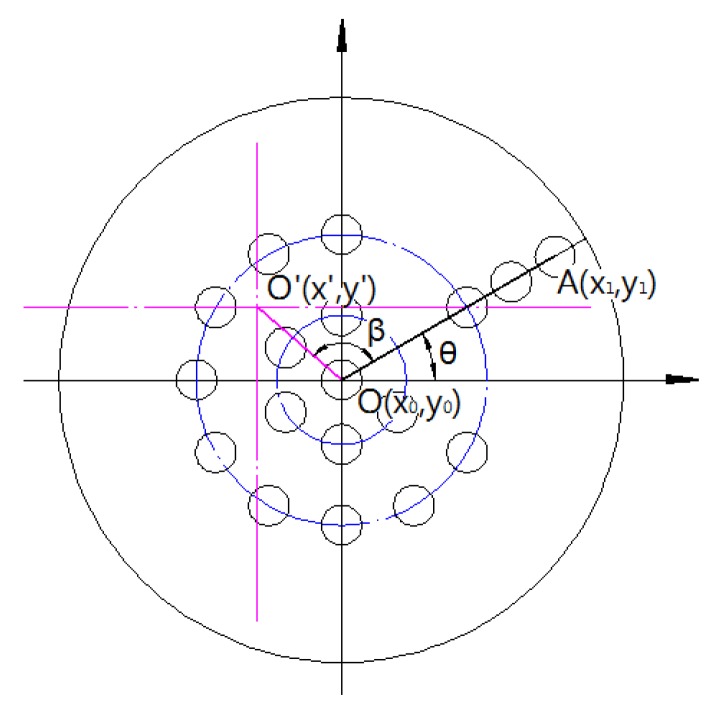
Schematic diagram of target for calculating deflection and angle. (The blue circles show the layout of LEDs. The red reference lines are to show the center of drill rod. The line *AO* shows the direction of drill bit while the line OO′ shows the deviation of it.).

To avoid visual evaluation of direction and to get precise guidance for operators, it is necessary to calculate the exact value of the face angle *θ* and rotation angle *β*, as shown in Figure 12.

The face angle *θ* can be described as two kinds of status in the expression (5).

(5) $$\left\{ \begin{array}{l} \theta >0,y_{1}-y_{0}>0\\ \theta \leq 0,y_{1}-y_{0}\leq 0 \end{array}\right.$$

It is calculated with the expression (6).

(6) $$\theta =\arctan\left|\frac{y_{1}-y_{0}}{x_{1}-x_{0}}\right|$$

From expressions (5) and (6), we obtain the face angle, *θ*, as the expression (7).

(7) $$\theta =\left\{ \begin{array}{l} \arctan\left|\frac{y_{1}-y_{0}}{x_{1}-x_{0}}\right|,y_{1}-y_{0}>0\\ -\arctan\left|\frac{y_{1}-y_{0}}{x_{1}-x_{0}}\right|,y_{1}-y_{0}\leq 0 \end{array}\right.$$

According to coordinates of point *O,* $\left(x_{0},y_{0}\right)$, point *A,* $\left(x_{1},y_{1}\right)$ and point *O′*, (x′,y′) the rotation angle, *β*, is calculated with the expression (8).

(8) $$\beta =\arccos\frac{\left[\left(x\prime -x_{0})\cdot (x_{1}-x_{0})+(y\prime -y_{0})\cdot (y_{1}-y_{0}\right)\right]}{\sqrt{{\left(x\prime -x_{0}\right)}^{2}+{\left(y\prime -y_{0}\right)}^{2}}\cdot \sqrt{{\left(x_{1}-x_{0}\right)}^{2}+{\left(y_{1}-y_{0}\right)}^{2}}}$$

The variate *V*, as an indicator of rotating direction, can be also calculated with the expression (9).

(9) $$V=(y\prime -y_{0})\cdot x_{1}+(x\prime -x_{0})\cdot y_{1}+(x_{0}\cdot y\prime -x\prime \cdot y_{0})$$

If V≥0, it means that the point *A* is on the right side of the line *OO′*, as shown in Figure 12. Operators could steer the drill bit counterclockwise with the angle of *β* to make line *AO* run through point *O′*. Otherwise, the drill bit rotates clockwise. 

### 4.4. Deflection Detection Algorithm

Drilling deflection is defined as the distance between the drill bit and the pre-defined track. In the image, it is the distance between the center of the LED target and center of the drill rod, or the length of *OO′*, as shown in Figure 11. The horizontal alignment is obtained by the expression (10).

(10) $$\Delta x=x_{0}-x\prime$$

And vertical alignment is denoted as the expression (11).

(11) $$\Delta y=y_{0}-y\prime$$

where $x_{0}$, x′, $y_{0}$, y′ are coordinates of points *O* and *O′*. The deflection is automatically calculated by the expression (12).

(12) $$L=\sqrt{{\left(x_{0}-x\prime \right)}^{2}+{\left(y_{0}-y\prime \right)}^{2}}$$

It is theoretically possible to calculate the deflection through multiplying L by the magnification of the CCD camera. However, in practice, the size of the image decreases with the length of drilling. For this reason, another algorithm is supplied to convert single pixel size to its real size in the target. Only when the magnification is calculated, can deflection in real be calculated and displayed on the interface.

### 4.5. Auto-Focus Algorithm

The LED target gets increasingly farther away from the camera as the drill bit advances. Thus, images captured by the camera become increasingly blurred. An operator is required to continuously tune the lens of the camera to adjust the focus distance and to maintain image clarity. However, manual focus requires an operator to keep watching on the camera. This, too, is a waste of manpower and fails to improve efficiency [18]. To achieve true auto-focus, a motor is employed to adjust the camera’s focus distance via a gear train. Thus, the relationship between the tuning angle and the object distance needs to be investigated. 

Images of the target are shot from the near to the distant. The angle tuned for lens increases as well. Concrete results are shown in Table 1. List 1 represents the object distance. List 2 shows the angle tuned for lens to improve focus at different distances. 

**Figure 13:**
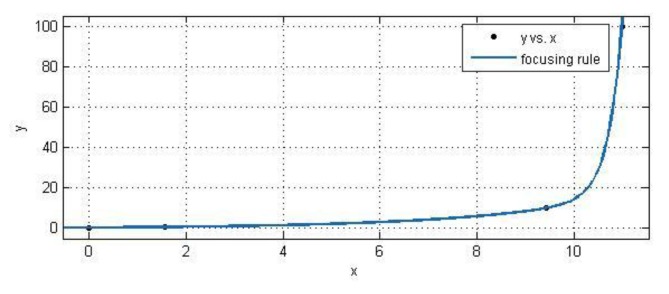
Curve of exponential fitting of focusing experimental results.

Results in Table 1 can be processed by CFTOOL, a curve fitting toolbox in MATLAB (R2017b, MathWorks^®^, Natick, MA, USA) [19,20]. Angles are independent variable, *x*, and observation distances are dependent variable, *y*. Drawing these points on an axis, it is easy to find that those points roughly obey the law of an exponential function. Therefore, exponential function, f(x)=a*exp(b*x)+c*exp(d*x), in MATLAB (R2017b, MathWorks^®^, Natick, MA, USA), is employed to fit them [21]. The fitted curve is then shown in Figure 13, wherein, a = 3.244 × 10^–15^, b = 3.437, c = 0.4169, and d = 0.3331. The focusing rules of the theodolite is denoted as the expression (13).

(13) $$y=3.224\times 10^{-15}\cdot e^{3.437\cdot x}+0.4169\cdot e^{0.3331\cdot x}$$

In the curve-fitting, there are two main indices to evaluate the fitting effect: the sum of squares for error (SSE) and the coefficient of multiple determination *R*^2^ [22]. SSE measures the deviation of the responses from their fitted values. A value closer to 0 indicates a better fit. R-square measures how successful the fit is in explaining the variation of data. A value closer to 1 indicates a better fit. In our fitting, SSE = 0.2617, *R*^2^ = 1, from which we can ensure that the focusing rules of the theodolite are reliable.

In this paper, the length of *OO′* is used as the criterion for the clarity of image. When the length is short (i.e., within a certain threshold), the image is considered “unclear,” so that focusing is required. Otherwise the image is clear enough, and no further focusing is needed. 

**Figure 14:**
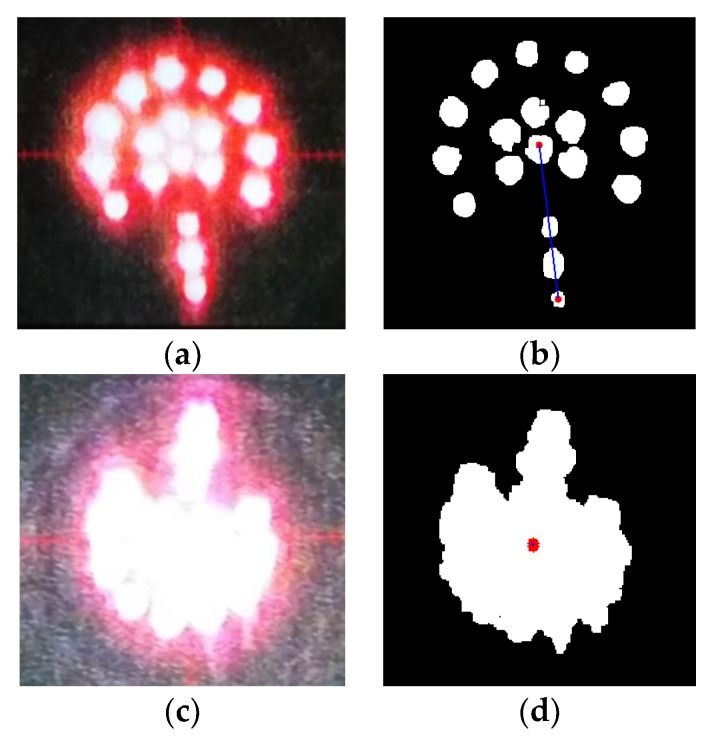
Results of different original images. (**a**) clear original image; (**b**) clear image after processing; (**c**) unclear original image; (**d**) unclear image after processing. (The above red dot in (**b**) means the center of the target image while the below is the center of the direction point. The blue line joining there two points are to show the direction of drill bit. The red dot in (**d**) means the center of target image calculated by software system.).

For instance, Figure 14b shows the result processed from Figure 14a. It clearly indicates the center and direction of the target. Currently, the distance between the center and direction point is relatively long, indicating that there is no need to focus. During manual estimation, the image of the LED target in Figure 14c is unclear. Although the proposed guidance system is still capable of calculating the center of the LED target, as shown in Figure 14d, the distance between the center of the LED target and direction point is too short. To avoid incorrect system judgment, a focusing operation is performed. 

## 5. Field Trial

**Figure 15:**
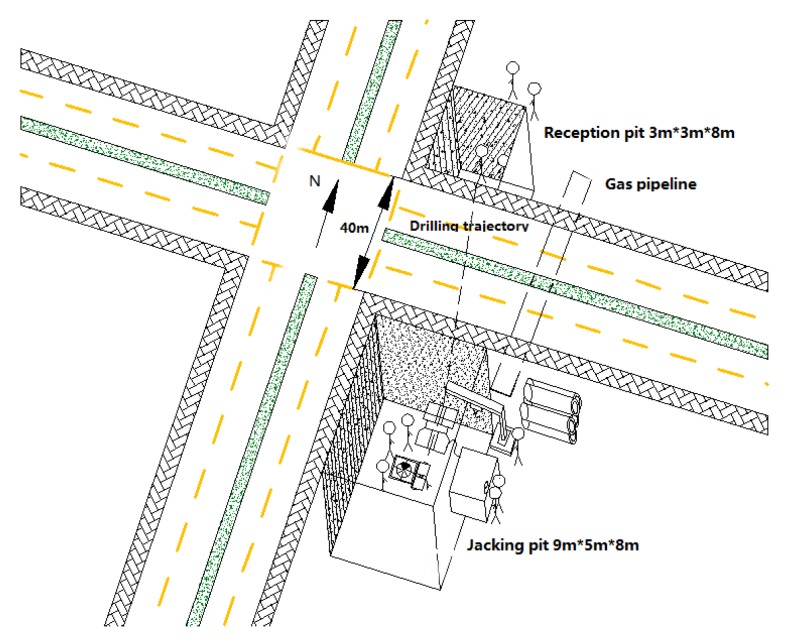
Schematic construction site.

The guidance system was used for an installation of hot water pipelines under Jin Ye Road and Zhang Ba Liu Road in Xi’an city, China. The contractor employed the HAB method because of its better adaption to soil conditions and convenience of removing spoil. In this project, a 40 m length and 800 mm diameter hot water pipeline was buried underground at a depth of 8 m. The inner diameter of the pilot pipe was 60 mm and the outer was 80 mm. The schematic construction site is shown in Figure 15.

The jacking and reception pits were excavated on both sides of the road in advance. The hot water pipeline was thrusted from the jacking pit to the reception pit. Workers set a theodolite-mounted a zoom CCD camera behind the power head of the auger boring machine to monitor the LED target.

**Figure 16:**
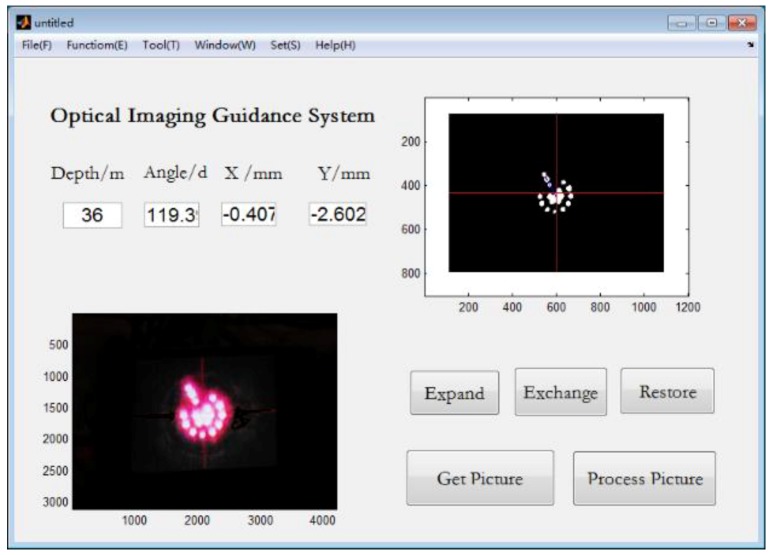
Interface of guidance system with results at 36 m.

Images of the LED target were transmitted to the personal computer as they were shot, every 6 m. Then, they were processed and calculated simultaneously. Figure 16 shows the interface of the guidance system with results obtained from the image at 36 m, including drilling length, horizontal and vertical alignments, and LED target angle. An operator steered the drill bit to change the drilling direction for correcting drilling trajectory according to these parameters. 

**Figure 17:**
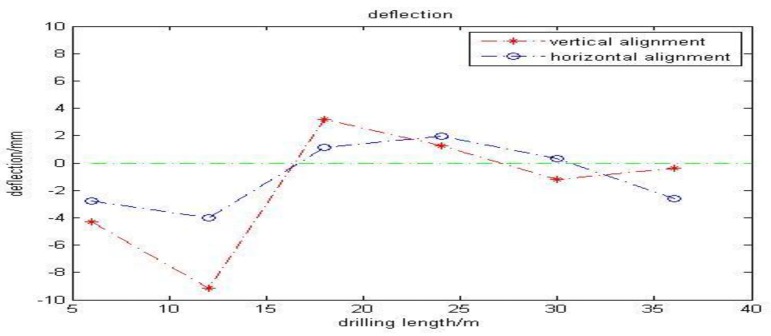
Line graph of deflections every 6 m. (The green line is the reference line meaning no deviation.).

The CCD camera captured a total of six images of the LED target during the drilling process. Table 2 displays horizontal and vertical alignments of the drill bit. More intuitively, Figure 17 illustrates the deflection in a line graph along with drilling length. The largest deflection emerged at 12 m, still within the acceptable range. After each image was processed, an operator checked the parameters and steered the drilling bit to correct the drilling track, especially when the deflection was relatively large. During the drilling, deflection gradually reduced. Using the enhanced automated guidance system, the operator carried a portable computer and mastered the drilling situation via the given parameters. The proposed guidance system thoroughly simplified the whole operation. Additionally, they avoided subjectively judging the deflection. 

Guided by the enhanced system, the pilot borehole was successfully drilled to the preset target area. The error between the excavated site and the destination was about 1.5 mm. The enhanced automated guidance system successfully completed the construction of a pilot bore hole. 

## 6. Conclusions

An enhanced automated guidance system, based on image processing for HAB, used in the installation of gravity or pressure pipelines, was proposed in this paper. This system consisted of a lighted target, theodolite, a camera with stand, and a personal computer, which acquired images of the lighted target, via a camera, and automatically calculated the deflection using image processing technology. To improve guidance quality, the emphasis of the research focused on the optimization of the LED light target and the enhancement of algorithms of deflection computing and correction.

To ensure target images were clear enough, multiple experiments were conducted to optimize the light color, filter plate color, luminous intensity, and LED target layout. LED targets are obtained with optimized key parameters, including red LED color, blue filter plate color, 11 mm spaces between LEDs, 160 Ω resistance, and two 1.5 V dry batteries. 

To analyze the images, preprocessing methods, including image enhancement, image sharpening, and image segmentation were implemented. Additionally, four algorithms (i.e., feature extraction, angle measurement, deflection detection, and auto-focus) were designed to accurately calculate the deflection and automatic camera focusing. 

The system was designed to guide the hot water pipeline installations. The accuracy was controlled within 2 mm over 48 m, meeting the installation requirement. The feasibility and reliability of this guidance system was also verified in a field trial. The guidance system is low-cost, and a small-workspace is required. The image processing technology and auto-focus method applied in this system promoted guidance automation, which freed machine operators from monitoring the screen all the time to estimate the deflection and to focus the target.

The indoor experiments showed that the proposed system can acquire clear images from about 100 m, meeting the requirements of general practice engineering. Then, the system was used to guide a hot water pipeline installation at the length of 40 m and a diameter of 800 mm, buried 8 m beneath the surface. The location error at the location of emergence was about 1.5 mm, meeting the installation requirement. This strongly verifies the feasibility and reliability of the proposed guidance system in practice.

## Figures and Tables

**Table 1:** Angles tuned for lens at different object distances on the verification experiment.

Distance (m)	Angle
0	0
1	π/2
10	3π
100	7π/2
+∞	4π

**Table 2:** Deflection values after processing images of LED target.

Type	Value					
Drilling length	6 m	12 m	18 m	24 m	30 m	36 m
Horizontal alignment (mm)	–4.299	–9.157	3.207	1.243	–1.223	–0.407
Vertical alignment (mm)	–2.759	–4.022	1.122	1.985	0.312	–2.602
